# Fluidic operation of a polymer-based nanosensor chip for analysing single molecules

**DOI:** 10.1017/flo.2022.8

**Published:** 2022-06-27

**Authors:** Swarnagowri Vaidyanathan, Sachindra Gamage, Kavya Dathathreya, Renee Kryk, Anishkumar Manoharan, Zheng Zhao, Lulu Zhang, Junseo Choi, Daniel Park, Sunggook Park, Steven A. Soper

**Affiliations:** 1 Bioengineering Program, The University of Kansas, Lawrence, KS 66045, USA; 2 Department of Chemistry, The University of Kansas, Lawrence, KS 66045, USA; 3 Department of Mechanical Engineering, The University of Kansas, Lawrence, KS 66045, USA; 4 Center of BioModular Multiscale Systems for Precision Medicine, Lawrence, KS 66047, USA; 5 Mechanical & Industrial Engineering, Louisiana State University, Baton Rouge, LA 70803, USA; 6 Department of Cancer Biology and KU Cancer Center, The University of Kansas Medical Center, Kansas City, KS 66106, USA

**Keywords:** Plastic nanofluidics, Hydrodynamic flow, Electrokinetic flow, *in vitro* diagnostics

## Abstract

Most medical diagnostic tests are expensive, involve slow turnaround times from centralized laboratories and require highly specialized equipment with seasoned technicians to carry out the assay. To facilitate realization of precision medicine at the point of care, we have developed a mixed-scale nanosensor chip featuring high surface area pillar arrays where solid-phase reactions can be performed to detect and identify nucleic acid targets found in diseased patients. Products formed can be identified and detected using a polymer nanofluidic channel. To guide delivery of this platform, we discuss the operation of various components of the device and simulations (COMSOL) used to guide the design by investigating parameters such as pillar array loading, and hydrodynamic and electrokinetic flows. The fabrication of the nanosensor is discussed, which was performed using a silicon (Si) master patterned with a combination of focused ion beam milling and photolithography with deep reactive ion etching. The mixed-scale patterns were transferred into a thermoplastic via thermal nanoimprint lithography, which facilitated fabrication of the nanosensor chip making it appropriate for *in vitro* diagnostics. The results from COMSOL were experimentally verified for hydrodynamic flow using Rhodamine B as a fluorescent tracer and electrokinetic flow using single fluorescently labelled oligonucleotides (single-stranded DNAs, ssDNAs).

## 1. Introduction

Point-of-care tests (POCTs) that use microfluidics and/or nanofluidics allow for disease assessment with attributes of timely results and full process automation to improve patient outcome (Vashist, 2017). In addition, POCTs using microfluidics/nanofluidics can reduce the costs associated with diagnosis and make even sophisticated tests more accessible to people in resource-limited environments. (Levit, Balogh, Nass, & Ganz, 2013) Challenges with translating POCTs into the field include designing platforms easily carried out by untrained operators, and being able to produce them in a high production mode with tight tolerances and at low cost (Dhawan et al., 2015; Shaw, 2015). Inorganic substrates such as glass, silicon or fused silica, have been the material of choice for micro- and nanofluidic devices (Sivanesan, Okamoto, English, Lee, & Devoe, 2005; Tas et al., 2002). The challenges associated with these materials include high fabrication costs, and the inability to scale up for mass production (Tas et al., 2002), especially when earmarked for molecular diagnostics.

Nanofluidics are particularly attractive for POCT applications because they can offer some unique measurement modalities that are not possible using microfluidics. For example, confinement of nucleic acids, such as double-stranded (ds) DNA molecules, in nanometre channels allow the dsDNA to undergo a conformational change from its natural coiled structure to a “rod-like” structure with stretching to different percentages with respect to its full contour length depending on the nanochannel geometry. Many applications have used DNA linearization in nano-dimensional channels for restriction mapping of DNA (Liu, Qu, Luo, & Fang, 2011), identification of methylation patterns and DNA damage (Yuan, Garcia, Lopez, & Petsev, 2007).

Several techniques have been reported for fabricating nanofluidic devices, including photolithography, electron beam lithography (EBL), focused ion beam (FIB) milling and nano-imprint lithography (NIL). Different substrates, including silica (Si), glass (SiO_2_) and polydimethyl-siloxane (PDMS), have been used with the appropriate fabrication methods (Wu, Chantiwas, Soper, & Park, 2010). Nanochannels fabricated using FIB or EBL have mainly been performed on materials like Si or SiO_2_ to achieve two-dimensional structures to ~10 nm. However, EBL and FIB have limitations such as high cost, low throughput and pattern limitations.

To increase the production rate of nanofluidic devices and thus increase their potential use for POCTs, thermoplastic-based nanofluidic devices are attractive. Thermoplastics are high molecular weight, linear or branched polymers with a lower Young’s modulus compared with inorganic material and possess a wide range of physicochemical properties. The deformability of thermoplastics makes them useful substrates for the fabrication of nanofluidic channels via replication techniques. Typical thermoplastics, including polymethyl methacrylate (PMMA), polycarbonate, cyclic olefin copolymer (COC) and polyethylene terephthalate, possess glass transition temperatures that are significantly lower than that of glass allowing for the fabrication of nanostructures at high production rates, low cost and high fidelity using techniques such as NIL. Furthermore, copolymers can be used as a substrate for nanofluidic devices that can extend the range of physiochemical properties arising from differences in the ratio of monomers used in the copolymer (Chou, Krauss, & Renstrom, 1995).

NIL is an effective technique to produce low-cost nanostructures with high resolution and was first developed in the 1990s (Chou et al., 1995; Chou, Krauss, & Renstrom, 1996). NIL uses fabricated micro/nanostructures patterned into Si for example via EBL or FIB to create a mould master from which structures can be imprinted (Chou et al., 1995). Structures down to ~5 nm are possible and NIL can be used with a wide range of polymer substrates. The main advantage of NIL is the ability to build multi-scale patterns in a single imprinting step (Liu et al., 2013).

The aforementioned technique for producing nanostructures in thermoplastics employs a top-down approach and, as such, requires an assembly step to enclose the fluidic network. This consists of bonding a cover plate to the substrate possessing the fluidic network, a process that typically involves heating the cover plate and substrate to a temperature near the glass transition temperature (*T*_*g*_) of the material for bonding of materials such as thermoplastics. This assembly step can involve thermal- or solvent-assisted fusion bonding (Xu et al., 2012). In a recent report, we bonded cover plates to nanofluidic substrates using a hybrid thermal fusion bonding process, which generated device process yield rates >90 %. The assembly process employed a thermoplastic substrate with high *T*_*g*_ thermally fusion bonded to a cover plate with a lower *T*_*g*_ (Uba et al., 2015b). Device assembly was achieved by bonding a plasma treated cover plate and substrate at a temperature ~5°C lower than the *T*_*g*_ of the cover plate. COC (*T*_*g*_ = 75°C) was used as the cover plate for a PMMA substrate.

**Figure 1.**
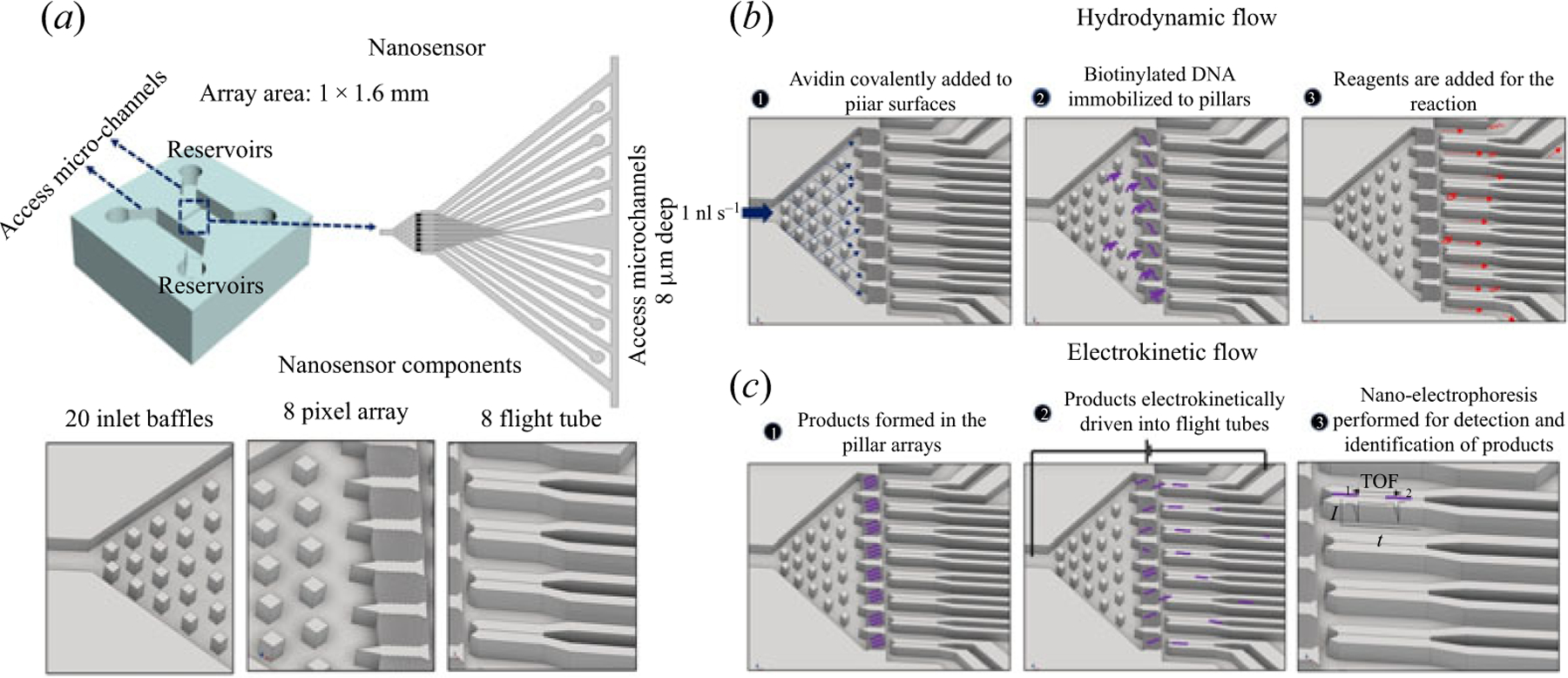
(a) Schematic of the nanosensor. The nanosensor is placed between two micrometre-sized access channels and is comprised of three main features; 20 baffles of 7 μm ×7 μm each to distribute reagents; 8-pillar arrays containing 268 pillars/array to perform solid-phase biological reactions; and 8 nano-tubes of 50 nm × 50 nm × 50 μm (w × d × l). (b) Sample processing steps carried out by the nanosensor that are enabled by hydrodynamic flow, which included pumping reagents (avidin) required for the solid-phase reactions (1); target DNA molecules immobilized onto the pillars of the array (2); and solid-phase reactions performed by adding the necessary reagents (3). (c) Electrokinetic flow is performed on the nanosensor chip and in the first step, the reaction is performed (1) followed by the products electrokinetically driven into the flight tubes through application of a DC voltage by placing electrodes at the end of the microchannel and grounding the ends of each nano-tube (2). Finally, once the products enter the nano-tube, they are detected using electrical measurements and the time-of-flight deduced for single DNA molecules to allow for their identification (3).

In this manuscript, we discuss the design, fabrication and, in particular, fluidic operation of a nanosensor (figure 1a) that is capable of detecting ssDNAs. This device was made from thermoplastics and contains nanostructures for ssDNA detection with microstructures integrated directly to the chip to allow for sample pre-processing directly within the mixed-scale chip. The nanosensor consisted of 8 pillar arrays, where a variety of surface-mediated reactions can be performed.

The pumping used in the nanosensor included hydrodynamic and electrokinetic. Hydrodynamic pumping was used to insert reagents into the nanosensor for a solid-phase molecular reaction. This included introducing reagents required for the solid-phase reaction and adding a target that binds to the pillars via an affinity association event. Following the solid-phase reaction, waste products were removed using hydrodynamic flow as well (figure 1b). Then, electrokinetic pumping was used to specifically shuttle the solid-phase reaction products into nanometre channels (i.e. nano-tubes). When the solid-phase product translocates through the nanochannel, detection can be performed to readout products generated from the solid-phase reaction and potentially identify them as well (figure 1c).

The nanosensor chip has some unique characteristics including: (i) implementation of both hydrodynamic and electrokinetic pumping in conjunction with fluidic nanochannels to provide passive valving; (ii) solid-phase sample preparation with nanochannel readout; (iii) mixed-scale fluidic network fabricated in a thermoplastic conducive to accommodate POCT for diagnostics; (iv) solid-phase reactions to allow for on-chip sample preparation; and (v) single-molecule readout.

## 2. Materials and methods

### 2.1. Fabrication of the nanosensor

The nanosensor master mould was made in silicon (Si) and contained both micro- and nanoscale features. The microscale features were fabricated using photolithography followed by etching the Si to ensure appropriate pattern transfer. For positive photolithography, AZ9260 resist was spin coated onto a Si wafer at a thickness of 8 μm and exposed to UV light (365 nm). After developing the resist, deep reactive ion etching (DRIE) was performed to etch the Si to the desired depth (7–8 μm) with the AZ9260 resist serving as the mask for the DRIE process. Further, the nanostructures on the Si wafer (pillar array region containing 1 μm pillars, 250 nm spacing between them and the nano-tubes of 50 nm × 50 nm) was fabricated by FIB milling using Ga ions. Structures were milled with 48 pA beam current and 1 μs dwell time. The milling conditions were optimized by iteration and validated with metrology.

### 2.2. Fabrication of the nanosensor in thermoplastic using NIL

Once the Si master was fabricated, patterns were transferred to a resin by adding 20 μl of a UV resin (poly-urethane acrylate) onto the Si wafer and placing a COC back plate over it and exposing the assembly to UV light (365 nm) for 2.5 min. The COC back plate containing the resin layer with structures was verified with the appropriate metrology such as SEM (channel width) and atomic force microscopy (AFM) (channel depth) to confirm proper dimensions of the desired structures. This was followed by thermal-NIL, where the resin stamp structures were transferred to a poly(methylmethacrylate), PMMA, thermoplastic substrate at 135°C, 300 psi, for 5 min. Following thermal NIL, a COC 8007 cover plate was treated with O_2_ plasma at 50 W for 1 min and thermal fusion bonded to plasma treated PMMA imprinted device at 70°C, 120 psi for 15 min.

### 2.3. Metrology of the Si master and thermoplastic substrate

After fabrication of the Si master, resin stamp and the thermoplastic device (i.e. before thermal fusion bonding of the cover plate to the imprinted substrate) were interrogated using SEM and/or AFM. The dimensions of the Si master, resin stamp and imprinted substrate were also verified using a Keyence VX-200 rapid scanning confocal microscope.

### 2.4. Micromilling of thermoplastic

The nanosensor possessed 8 nano-tubes associated with each pillar array. The nano-tubes were extended into microchannels with the ends of the microchannels terminated with reservoirs that were 60 μm in diameter. These reservoirs were drilled using a micro-milling machine as a through hole in the thermoplastic substrate with the bottom of the through hole sealed using a COC cover plate. A Pt electrode connection was placed in each reservoir, which allowed application of a DC voltage across each nanochannel to perform electrokinetic pumping.

### 2.5. COMSOL

We built a two-dimensional nanosensor model in AutoCAD and tested different flow patterns using finite element analysis simulations (COMSOL 5.5) for both electrokinetic and hydrodynamic flows. The electrolyte used for the simulations was 1× tris borate EDTA (TBE) at a flow rate of 1 nl s^−1^ for hydrodynamic pumping. The physics used was laminar flow for hydrodynamic flow and electrostatics and transport of dilute species for electrokinetic flow. A no-slip condition was used for the boundary conditions as the Knudsen number was well below 0.01 (continuum flow).

### 2.6. Hydrodynamic flow

Rhodamine B, a neutral dye and soluble in water with excitation and emission wavelengths of 534/570 nm served as the tracer to track fluidic operation of the nanosensor using hydrodynamic pumping. One μM of Rhodamine B was hydrodynamically pumped at a flow rate of 1 nl s^−1^ using a syringe pump. The fluorescence from the dye flowing into the nanosensor was captured using a sCMOS camera at an exposure time of 10 ms using a 60× objective fitted into an inverted microscope. Images were collected and the intensity quantified using ImageJ. The mean intensity of each pillar array (calculated from 5 different experiments using the same concentration of the tracer dye) was calculated.

### 2.7. Electrokinetic flow

A 25 base single-stranded oligonucleotide labelled with Cy3 at its 5′ end (excitation/emission wavelengths of 534/570 nm) was used as a surrogate of solid-phase DNA products to allow tracking into the nano-tube (i.e. electrokinetic pumping). A 10 V DC potential was applied to the inlet with the terminal ends of the nano-tubes placed at earth ground. Single-molecule fluorescence tracking was performed and the electric field was turned off once the oligonucleotides reached the terminal end of the nano-tubes. The images were captured at 10 ms using a fluorescence microscope equipped with a 100X 1.4 NA oil immersion objective and a sCMOS camera.

### 2.8. Data analysis

Fluorescence images were collected from the sCMOS camera as a TIFF file, which was subsequently analysed using ImageJ. The fluorescence signal was represented quantitatively as line plots, which displayed grey-scale values of the area selected within the imaging area. The difference in grey values indicated differences in the fluorescence intensity between different areas. Experiments were repeated 5 times within the same area of the chip selected from which fluorescence intensity plots were generated. The difference in fluorescence intensity between subsequent trials representing either different conditions or areas of the chip were compared and statistical analysis carried out using a *p*-test. A *p*-value is a statistical measurement used to validate a hypothesis against observed data.

## 3. Results and discussion

### 3.1. Fabrication of the multi-scale nanosensor

The nanosensor chip contained both micro- and nanoscale structures all of which were used to affect the operation of the chip. The footprint of the nanosensor device was 1 mm × 1.6 mm. The device featured 20 baffles (7 μm × 7 μm) at the entrance, which were used to uniformly distribute the input to the 8 pillar arrays, with each array measuring 20 μm × 20 μm and containing 268 pillars. Each pillar was designed with a diameter of 1 μm and spacing of 250 nm. These pillars could act as a support for solid-phase reactions. The pillar number and spacing were selected to provide high surface area to increase the load of surface immobilized targets and increase capture efficiency of targets when flowing hydrodynamically through the pillar array. For example, at a volume flow of 1 nl s^−1^, the capture efficiency of targets as deduced from simulation was ∼80 %. Adjacent to each pillar array was a nano-tube that was designed to be 50 nm × 50 nm (w ×d) and 50 μm in length, and used to detect and/or identify solid-phase reaction products.

We used a top-down fabrication approach to produce the thermoplastic device, including NIL (Reisner et al., 2005; Weerakoon-Ratnayake, Vaidyanathan, Amarasekara, Johnson, & Soper, 2019). NIL is attractive because it can be used to fabricate the device in a medium-scale production mode as opposed to direct writing using EBL or FIB milling as typically done for inorganic substrates (Weerakoon-Ratnayake et al., 2019). Finally, the use of a thermoplastic with a low *T*_*g*_ allows structures to be replicated with high fidelity (Matschuk & Larsen, 2012). The imprinted thermoplastic substrate was sealed with a lower *T*_*g*_ cover plate compared with the substrate via thermal fusion bonding to minimize structure deformation (Uba, Hu, Weerakoon-Ratnayake, Oliver-Calixte, & Soper, 2015a). Basically, the fabrication process employed herein involved the fabrication of a Si master from which resin stamps were produced via UV-NIL followed by the production of the final device in a thermoplastic via thermal NIL (Kooy, Mohamed, Pin, & Guan, 2014; Tsao & Devoe, 2009; Weerakoon-Ratnayake, O’Neil, Uba, & Soper, 2017).

Supplementary information (SI) figure S1a (available at https://doi.org/10.1017/flo.2021.16) represents the process scheme used for the fabrication of the Si master; photolithography was used for fabrication of the inlet baffles (7 μm × 7 μm), and microchannels. Following photolithography, the Si wafers were etched using DRIE producing a feature depth of 6–7 μm (SI figure S1b). Then, FIB was used to make the 8 pillar arrays and nano-tubes. This design offered each pillar array with a high surface area (1242 μm^2^ array^−1^). SI figure S1c(i) shows an SEM image of the Si master with the FIB-milled pillar arrays and nano-tubes. The average size of the pillars in the Si master was 974 ± 8 nm with a spacing of 227 ± 12 nm (SI figure S1Cii). The nano-tubes (Supplementary figure S1Ciii) were found to be 66 ± 12 nm in width and depth. The terminal ends of the nano-tubes were connected to microchannels having individual reservoirs (60 μm in diameter). This was adopted so that a DC voltage could be applied by placing electrodes within these reservoirs.

**Figure 2.**
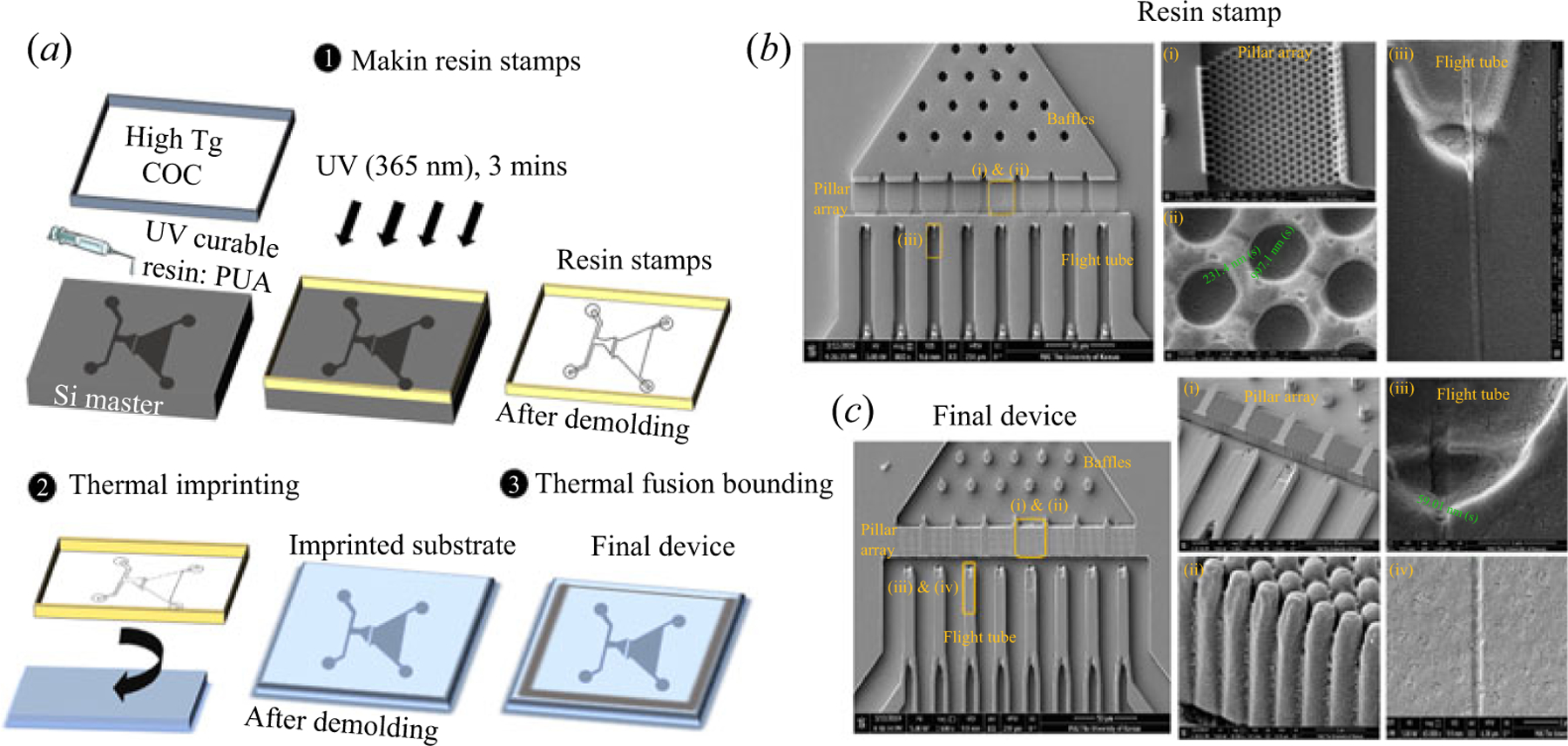
(a) Schematic of the fabrication and assembly steps of the nanosensor, which consists of 3 steps: (1) creation of resin stamps from the Si master by UV-NIL; (2) imprinting (thermal NIL) of the PMMA substrate using the resin stamp at 135°C, 300 psi, and for 5 min; and (3) thermal fusion bonding of a COC 8007 cover plate to the fluidic substrate at 70°C and 120 psi for 15 min. (b) SEMs showing the UV resin stamp where the structures were formed from the Si master. The pillar arrays (a) and their individual pillars (b), and the nano-tubes (c) were transferred into the PMMA substrate. (c) SEMs of the final PMMA device after thermal imprinting that has structures similar to the original Si master. The final device with the pillar arrays (a), the side profile of the pillars (b), the funnel entrance to the nano-tubes (c) and the nano-tubes (d).

After the pattern was successfully transferred to the Si master, the master was silanized using trichloro(1H,1H,2H,2H-perfluorooctyl) silane to make the surface hydrophobic for UV-NIL, which generated resin stamps (see figure 2a(1)). For UV-NIL, patterns were transferred from the Si master to a COC back plate covered with a resin that was placed against the Si master and cross-linked via UV-NIL. The pattern from the resin stamp was transferred to a thermoplastic using thermal NIL (figure 2a(2)) and, finally, thermal fusion bonding was used to enclose the fluidic network using a cover plate (figure 2a(3)). Figure 2(b) shows SEM images of the resin stamp. Figure 2(c) shows SEMs of the final device thermally imprinted into PMMA. It can be seen that the baffles, pillar arrays and the nano-tubes were successfully transferred into the PMMA substrate.

### 3.2. COMSOL simulations

**Figure 3.**
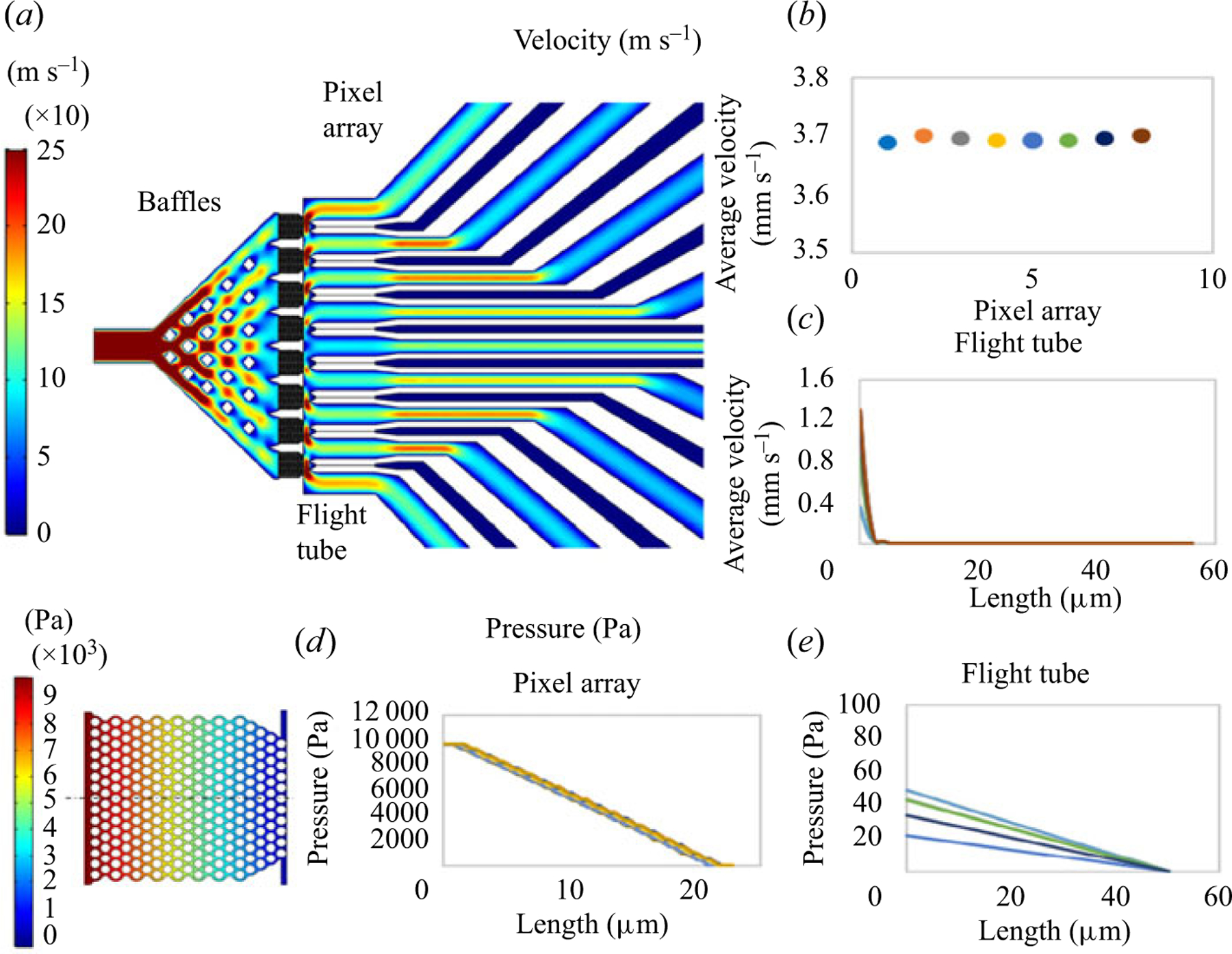
(a) COMSOL simulation of hydrodynamic flow of 1× TBE through the nanosensor device at a flow rate of 1 nl s^−1^. The entrance of the nanosensor (25 μm) is used as the common inlet of the sampling/reagents. The flow entering the nanosensor is distributed uniformly across the pillar arrays by the baffles such that all 8 pillar arrays receive a constant delivery of the input fluid. (b) Graph showing the simulated average velocity across the 8-pillar arrays, which is ~3.7 mm s^−1^. The velocity across the 8 pillar arrays possessed a standard deviation of 6 μm s^−1^ between the arrays. (c) Hydrodynamic flow across the 8 nano-tubes, where the flow was found to be negligible. This is empirically represented in the line graph that is drawn across the flight tubes, where the velocity at the entrance of the nano-tubes dropped to ~0 mm s^−1^. (d) Pressure drop across the pillar arrays, where the average pressure drop among the 8 pillar arrays were found to be similar. (e) The pressure drop across the 8 nanochannel nano-tubes with respect to the pressure drop seen in the microchannels that are between the nano-tubes.

A model nanosensor design was built in AutoCAD and flow patterns were evaluated using finite element analysis simulations (COMSOL). The device operation was simulated with 1× TBE buffer. For fluid flow driven hydrodynamically at a flow rate of 1 nl s^−1^, the placement of 20 diamond shaped chevron baffles in the entrance of the nanosensor facilitated distribution of fluid across the 8 pillar arrays. A uniform distribution of input fluid was necessary to ensure that reagents and targets reached all 8 pillar arrays. Each array was 20 μm × 20 μm spaced 5 μm from each other. The laminar flow module governed by the Navier–Stokes equations were used to model the flow of fluid under the influence of hydrodynamic pumping. For electrokinetic pumping, the electrostatics module (AC/DC physics) governed by the Poisson and Maxwell equations were chosen to model the electric field in the nanosensor by application of 10 V at the common input and placing earth ground at the outlet end of each nanoscale tube. Figure 3(a) shows the overall profile of the COMSOL simulated hydrodynamic flow through the nanosensor. It can be seen that the flow entering through a common input was equally distributed by the baffles to all pillar arrays (figure 3a).

The fluid leaving the pillar arrays preferentially entered areas surrounding nano-tubes (the red/orange areas). This indicated that during hydrodynamic pumping, fluid does not travel into the nano-tubes due to their high fluidic resistance; the pressure drop in the nano-tubes was calculated to be 35 psi, whereas the fluidic resistance in the channels between the nano-tubes was >3 psi. This indicates that generating channels with small cross-sections surrounded by larger cross-section channels can be used to minimize fluid flow into nano-tubes when using hydrodynamic pumping (i.e. passive valving).

In figure 3b, the average velocity across the entire array suggested that they were similar (~3.7 mm s^−1^), indicating uniform access of the input fluid to all pillar arrays; each pillar array received 16% of the total input fluid. SI figure S3 shows a vertical and horizontal line profile of fluid flow across a single pillar array. The uniformity of the flow in a single row of pillars was determined in both the horizontal (SI figure S3a) and vertical (SI figure S3b) directions, which also ensured target and reagent delivery to the pillars of all 8 arrays simultaneously. A magnified image of the flow profile (figure 3c) in one nano-tube showed that there was negligible flow in these nano-tubes during hydrodynamic pumping. There was a drop in velocity from 1.2 mm s^−1^ just prior to the nano-tube to ~0 mm s^−1^ through the length of the channel for all nano-tubes. This result was due to the high fluidic resistance in the nano-tubes with respect to the interstitial space between these tubes. Figures 3d and 3e show the pressure drop across the pillar arrays and nano-tubes, respectively.

**Figure 4.**
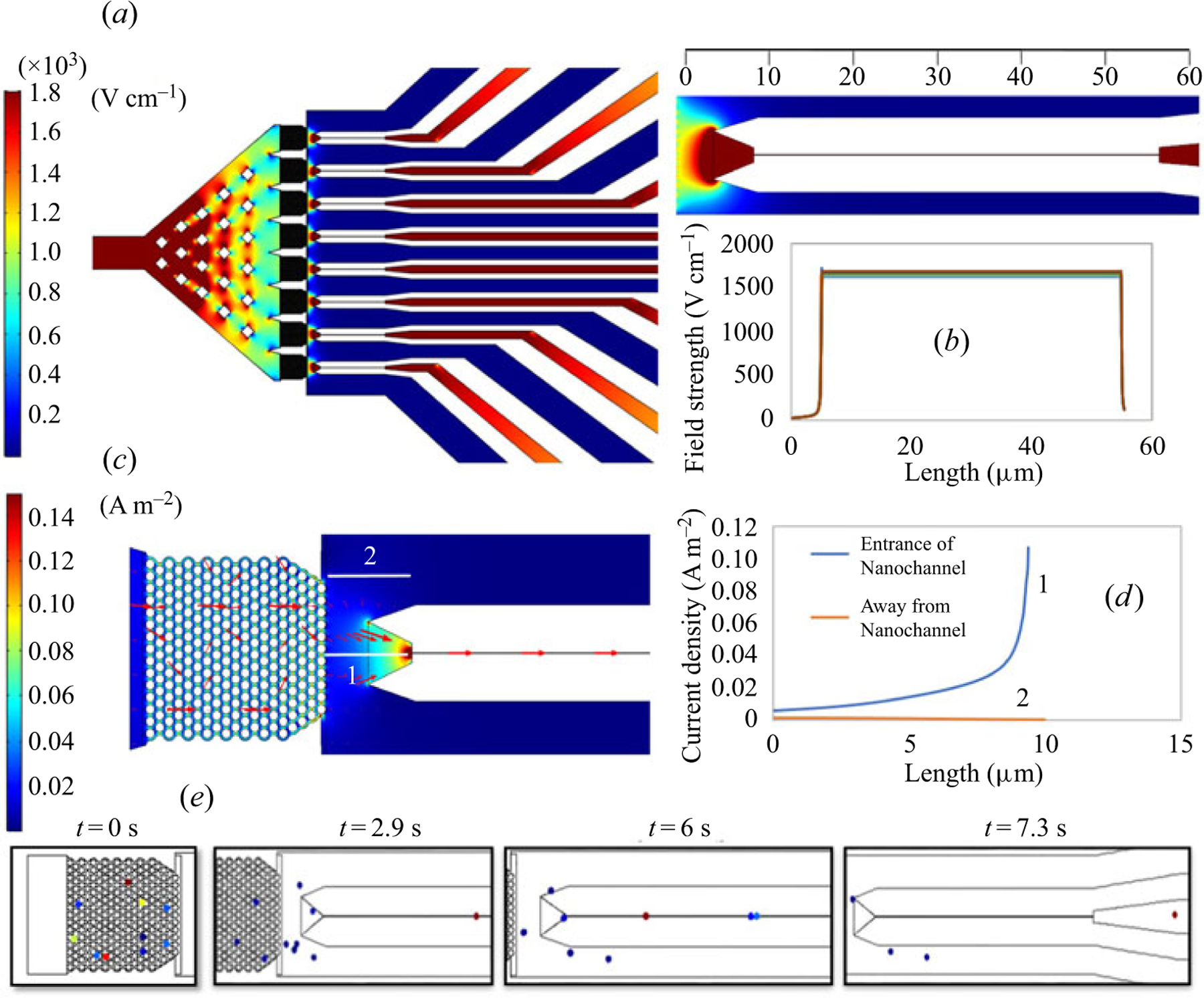
COMSOL simulation of electrokinetic flow through the nanosensor device. (a) Representation of the electric field strength across the nanosensor. A potential of 10 V was applied at the inlet of the nanosensor and each of the nano-tubes were placed at earth ground in their respective terminal reservoirs. The areas of the nanosensor having the smallest dimensions were found to have the highest electric field strength due to their higher fluidic resistance. (b) Contour and line plots of the electric field strength across the 8 nano-tubes. The electric field strength increased from the entrance and decreased at the output end and remained fairly constant through the majority of the nano-tube length. There was a similar electric field strength across the 8 nano-tubes, which was 1650 V cm^−1^. (c) Current density plot showing one pillar array and the nano-tube with arrows representing the direction of fluid movement and the length of arrows representing the field strength. (d) Line graph showing the current density up to 0.1 A m^−2^ at the entrance of the nano-tube and a drop in the current density to ~0.0015 A m^−2^ in the regions away from the nano-tube. (e) Particle tracing representative of ssDNA movement of 10 particles simulated in the pillar array (t = 0). The particles were observed to move preferentially into the nano-tube with more particles entering into the funnel (t = 2.9 s), and all of them translocating into the nano-tubes (t = 6 s). Those particles that are not in the funnel, but still in the radius of high current density were also drawn into the nano-tube (t = 7.3 s). The transfer efficiency (pillar array to nano-tube) was 80 % as deduced from the simulation results.

In the case of electrokinetic pumping, the goal was to selectively shuttle solid-phase reaction products into the nano-tubes for identification and detection. A potential was applied across the nanosensor by placing the inlet microchannel at −10 V and the ends of the individual nano-tubes at earth ground. The field strength across the entire nanosensor is shown in figure 4a. The areas of the sensor having a high electric field strength were the entrance of the nanosensor, the pillar arrays and the nano-tubes. A field strength of 1680 V cm^−1^ (i.e. potential drop of ~8.5 V observed in each nano-tube, see SI figure S4) across the nano-tubes was observed (figure 4b) with the remaining drop occurring across the pillar arrays (0.5 V) and the baffle region (SI figure S4a). A magnified image of a nano-tube showed a higher field strength at the entrance of the tube and constant drop continuing through the length of each nano-tube. There was a similar field strength profile across all 8 parallel nano-tubes.

The current density profile indicated that the direction of charged analyte movement would be preferentially from the pillar array to the nano-tube (size of the arrows represents the magnitude of the current density, figure 4c) and not the larger channels placed between each nano-tube. The line graph of the current density with arrows showed a higher current density (0.1 A m^−2^) at the entrance of the nano-tubes (figure 4c,d, line 1) compared with the output end. A line graph at an area away from the entrance of the nano-tubes indicated that there was a current density of 0.0015 A m^−2^ eventually dropping to ~0A m^−2^ at regions further away from the entrance of the nano-tubes (figure 4c,d, line 2).

Particle tracking simulations were next performed using ~1000 different oligonucleotides (i.e. particles) and the results indicated that ~80 % of the oligonucleotides entered the nanometre flight tubes. Representative particle tracing results for 10 events are shown in figure 4e for oligonucleotides entering the nano-tube from the pillar array under the application of a −10 V DC. The time evolution of particle movement (figure 4e) was: (i) *t* = 0 s, where the particles were in the pillar array; (ii) *t* = 2.9 s, where the first particle was seen inside the nano-tube; (iii) *t* = 6 s, where more particles were seen entering into the nano-tube; and (iv) *t* = 7.3 s in which most particles travelled through the nano-tube. The inlet of the nano-tubes had a funnel shaped entrance (see figure 2c showing the entrance of the nano-tube) tapering in width and depth leading to the nano-tube so that there was a larger capture area for products released from the pillar arrays (Vaidyanathan et al., 2021). The field strength profile (figure 4b) also showed that the area surrounding the funnel entrance had a small radius with a higher field strength.

### 3.3. Hydrodynamic pumping monitored using a fluorescent tracer

**Figure 5.**
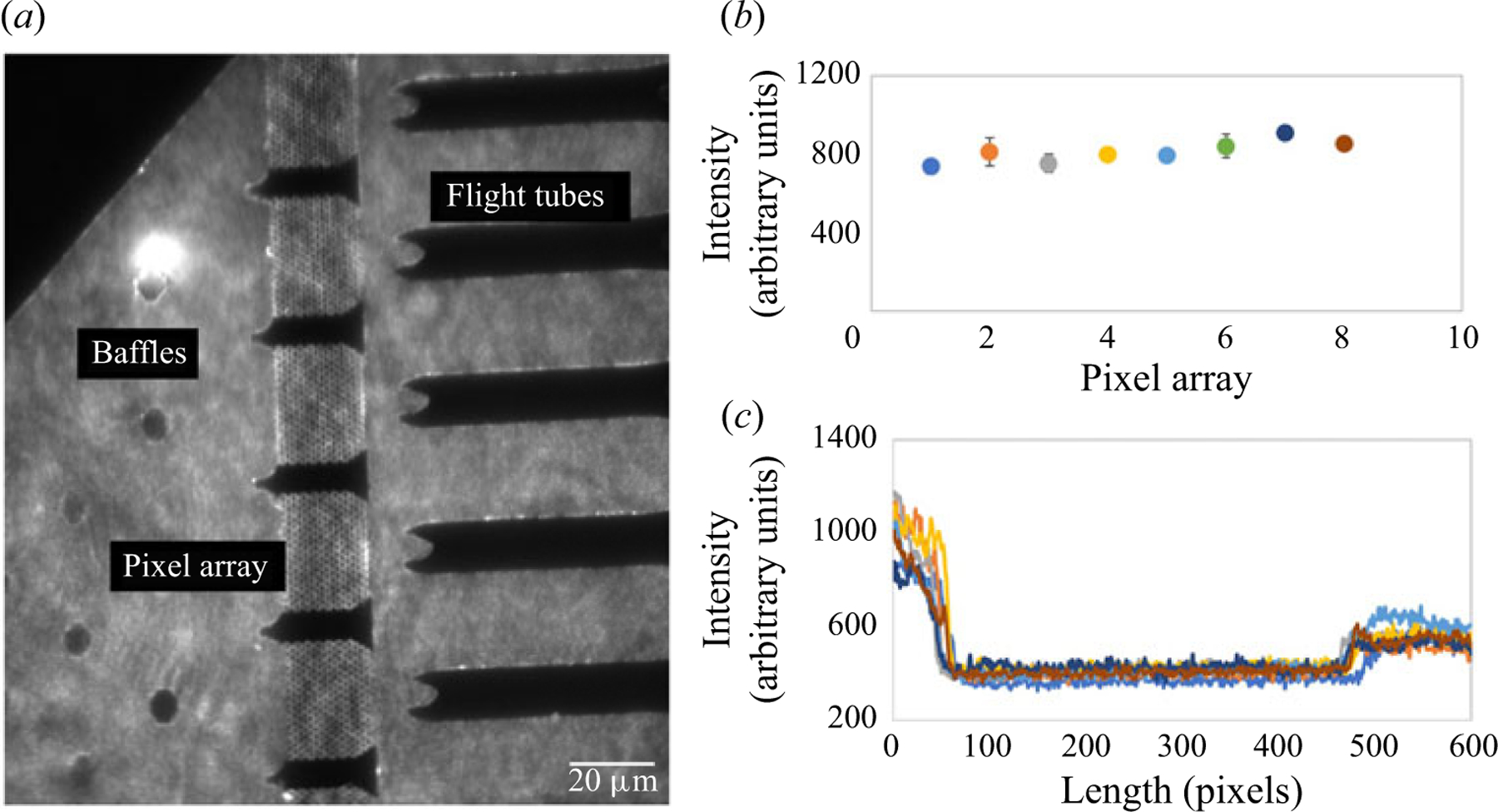
Experimental results of the hydrodynamic flow across the nanosensor. (a) Distribution of Rhodamine-B with hydrodynamic flow at a flow rate of 1 nl s^−1^. The dye was distributed across the pillar arrays by the baffles and preferentially into the microchannel areas surrounding the nano-tubes. No visible fluorescence was seen in the nano-tubes. (b) Fluorescence intensities across the 8 pillar arrays showing similar levels of intensities ranging from 800–950 (arbitrary units). The intensities were represented as grey-scale values. (c) The fluorescence intensity of the dye tracer was also evaluated in the nano-tubes, and a significant intensity drop was seen in the 8 nano-tubes. Areas having dye had a grey value >800, while the intensity in the nanochannels were ~300. The measurements were taken from 5 different experiments and used to calculate the mean and standard deviation. No statistically significant difference was observed between the intensity in the pillar arrays (p < 0.01 at 95 % CI). A scale bar of 20 μm is represented in the images. The fluorescence was captured using a sCMOS camera at an exposure time of 10 ms with the fluorescence collected using a 100× oil immersion objective. For the fluorescence measurements, the fluorescence intensity from the image in the presence of dye was measured in arbitrary units (14-bit camera setting; 16 384-grey-scale image) from the imaging camera and the background signal, also measured in arbitrary units with the same grey scale, was subtracted from the dye measurements.

We verified the simulation results for hydrodynamic pumping by using a fluorescent tracer in the carrier buffer and fluorescence microscopy. Rhodamine B, a neutral dye, with excitation/emission maxima of 534/570 nm was used as the tracer. The volume flow rate used was 1 nl s^−1^. An example image can be seen in figure 5a, which shows the distribution of dye tracer within the nanosensor at steady state. The dye front was traced during hydrodynamic flow, which showed that the dye moved around the baffles and was uniformly distributed to the pillar arrays. Subsequently, the dye moved predominately around the nano-tubes and entered the access microchannels. We observed only negligible amounts of dye in the nano-tubes. The mean intensity of each pillar array fluorescence image (calculated from 5 different experiments) were similar and agreed with the results seen in the COMSOL simulations (figure 5b). The intensity was represented as grey-scale values and ranged from 800–950 (arbitrary units) for all pillar arrays. The absence of fluorescence seen in the 8 nano-tubes are shown by the line graph in figure 5c and indicated minimal if any dye within the nano-tubes when pumping using hydrodynamics due to the high fluidic resistance in these channels compared with the access microchannels. The grey-scale values ranged from 200–250, which is almost 4 times lower than the fluorescence intensity observed in the pillar array. In terms of nanosensor operation, during loading of targets and reagents, there was equal accessibility of fluid to the arrays (demonstrated by both COMSOL and experiments; figure 5b). However, there was preferential movement of materials into microchannels between each of the nano-tubes due to their small fluidic resistance (figure 5c).

### 3.4. Monitoring electrokinetic pumping using fluorescently labelled oligonucleotides

**Figure 6.**
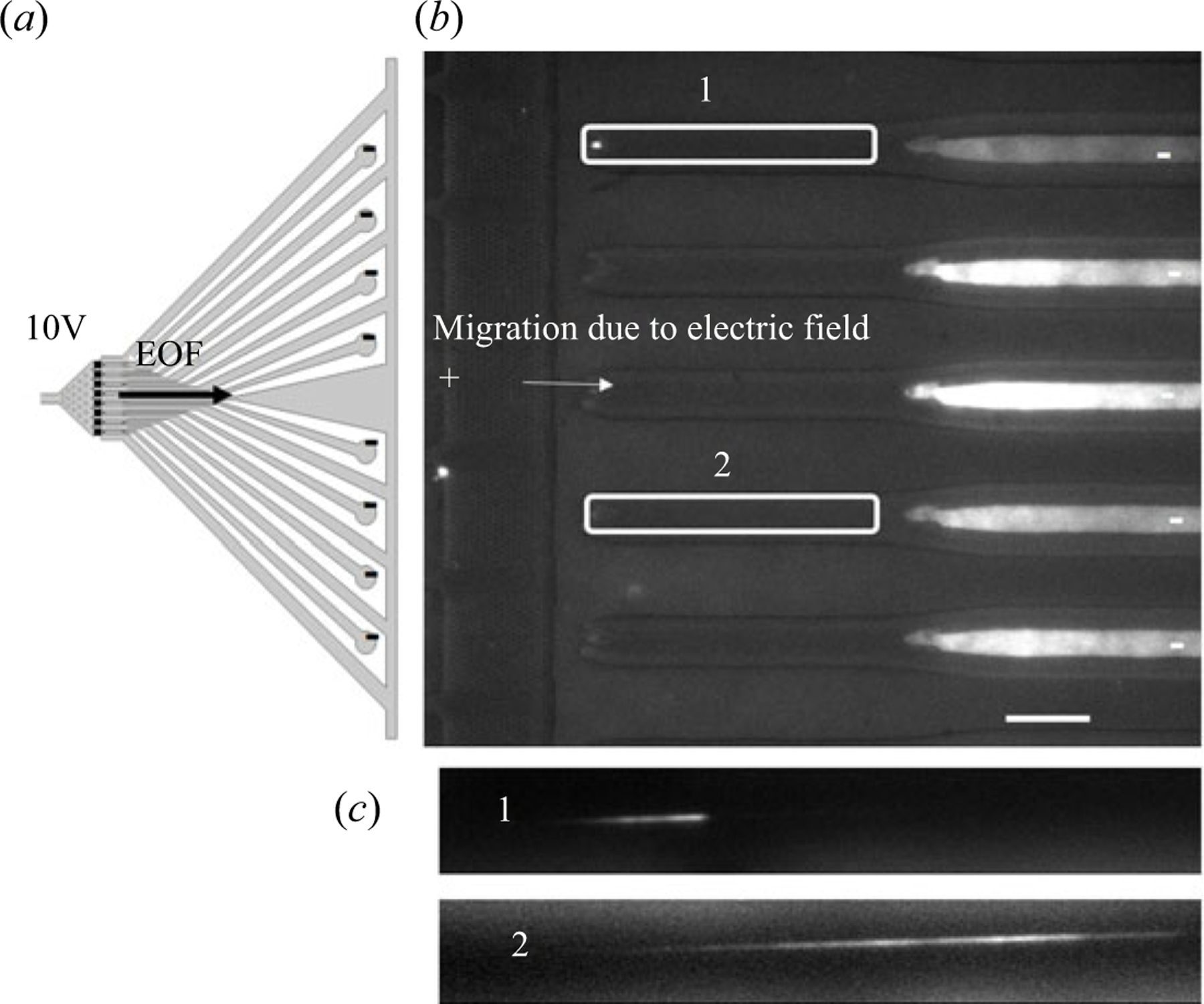
(a) Schematic of the application of the DC bias voltage across the nanosensor. The 10 V potential was applied at the microchannel entrance to the nanosensor, while the ends of the nanochannel nano-tubes were placed at earth ground. (b) Fluorescent images showing the accumulation of fluorescently labelled ssDNA (25 nucleotides in length) from the pillar array to the end of each of the nano-tubes, but not in the microchannels flanking each nano-tube. (c) The nano-tube images were collected using a sCMOS camera with ROIs 1 and 2 showing a single ssDNA molecule in the nano-tube. A scale bar of 20 μm is represented in the images. The applied voltage was −10 V at the inlet with 100 mW laser intensity (532 nm excitation) and a sCMOS gain of 3 with 1 × 1 binning. The sCMOS camera was operated in a 14-bit mode.

In the case of electrokinetic pumping, fluorescently labelled DNA oligonucleotides (ssDNAs) were electrokinetically injected into the nanosensor so that they could be tracked using single-molecule fluorescence microscopy. Here, we used Cy3-labelled 25mer oligonucleotides (random sequence) with the electrokinetic flow driven using −10 V. Once the potential was applied, the dye-labelled oligonucleotides were observed to move towards the cathode (figure 6a) due to the high electroosmotic flow (2.2 × 10^−4^ cm^2^ Vs^−1^) (Amarasekara et al., 2020) associated with the O_2_ plasma treated plastic that can generate surface confined −COOH groups (surface charge = −40.5 mC m^−2^) (Uba et al., 2015b). An advantage of using thermoplastics as opposed to inorganic substrates for electrokinetic pumping is that the plastic possesses an electroosmotic flow that can be controlled by the dose of UV/O_3_ exposure to the plastic (Uba, Hu, Weerakoon-Ratnayake, Oliver-Calixte, & Soper, 2015a; Uba et al., 2015b; Vaidya, Soper, & Mccarley, 2002). Figure 6b shows fluorescence intensity profiles confirming the presence of oligonucleotides being directed specifically into the nano-tubes. The intensity of these peaks ranged 300 to 550 arbitrary units compared with the background intensity value of 200 arbitrary units. This indicated during electrokinetic pumping, particles (i.e. ssDNAs) preferentially enter into the nano-tubes due to the larger electric field drop in these tubes (see figure 4). High exposure (3500 ms) region of interest (ROI) images showed fluorescence in two of the nano-tubes (regions 1 and 2) indicating the presence of oligonucleotides in these nano-tubes. The presence of oligonucleotides within the nanometre nano-tubes with the absence of fluorescence in the area between the nano-tubes indicated that electrokinetic pumping preferentially shuttled the oligonucleotides into the nano-tubes. The preferential transport of oligonucleotides into the nano-tubes compared with the microchannels was a direct result of the large electric field drop in the nanochannels with respect to that in the adjacent microchannels (figure 4). Thus, high efficiency transfer of charged products generated in the pillar arrays into the nano-tubes can be accomplished using electrokinetic pumping.

## 4. Conclusions

We have developed a mixed-scale nanosensor device made in a thermoplastic via NIL. While several operational steps are required for the operation of the nanosensor chip, we were able to show in this paper that, using a combination of hydrodynamic and electrokinetic pumping, passive valving was possible using dimensional features associated with different domains of the nanosensor. For example, during target loading and reagent introduction for solid-phase reactions, hydrodynamic pumping could be used to minimize introduction of material into the nano-tubes, which may cause device failure due to clogging or non-specific adsorption. Conversely, following solid-phase reactions successfully formed products can be selectively injected into the nano-tubes for interrogation using electrokinetic pumping due to the fact that the majority of the electric field drop occurs in these tubes. Particle tracking indicated that ~80 % of negatively charged molecules could be effectively inserted into the nano-tubes when the flow was actuated electrokinetically.

This device has the potential to perform sample preparation directly on chip followed by nanofluidic detection and identification of the products formed as a result of a solid-phase reaction, which can be used to detect unique sequences in both DNA and RNA that possess high diagnostic value for a number of diseases. In addition, these solid-phase reactions can quantify the expression of certain gene fragments (i.e. mRNA) following reverse transcription based on single-molecule counting. The solid-phase reactions can be carried out on 8 separate pillar arrays, which were comprised of 268 1-μm pillars, positioned adjacent to a nanometre tube to detect and identify successfully formed products. As an example, solid-phase ligase detection reactions can be performed, which we have already demonstrated for analysing single point mutations (Sinville et al., 2008) and then, utilizing single-molecule nanoscale electrophoresis for identifying reaction products based on differences in their size (Amarasekara et al., 2020).

**Figure 7.**
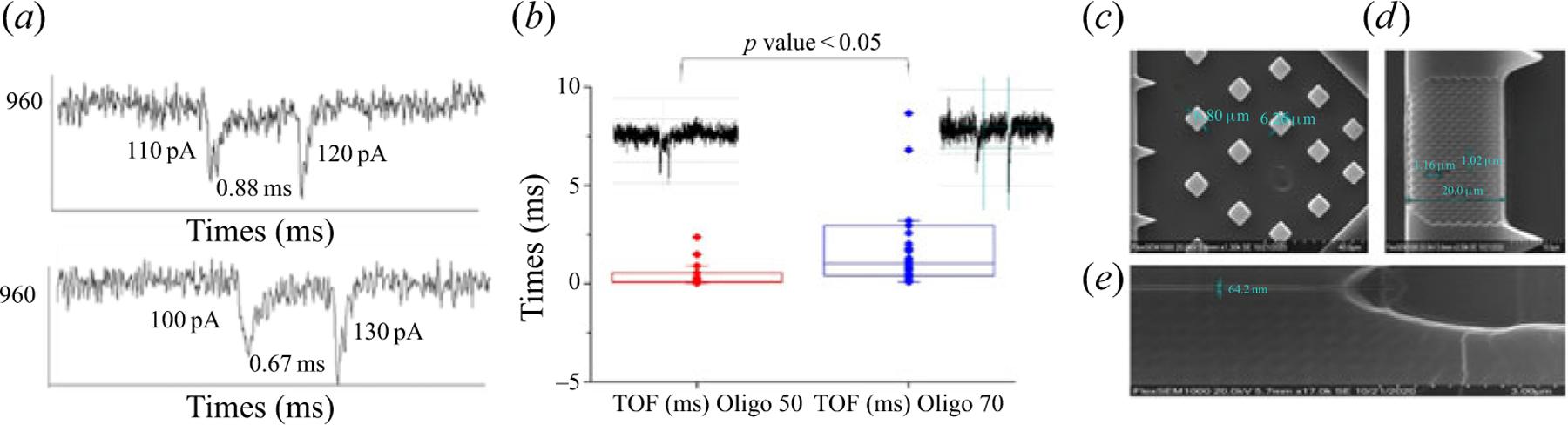
(a) Representative traces of oligonucleotide 70 showing two peaks as they pass through the two nanopores placed in series, having a TOF of 0.88 and 0.67 ms. The numbers listed (pA) represent the current transient amplitude. (b) Difference in TOF between an oligonucleotide with 50 nt and one with 70 nt (p < 0.05). (c–e) SEMs for nano-injection moulding of the nanosensor. The three regions shown are the baffle region (c), The pixel array with the pillars are shown in region (d), which was comprised of 268 1-μm diameter pillars that were 5 μm tall, the nano-flight tube which is 50 nm width and depth and 50 μm long are shown in (e).

Our future efforts will include incorporating solid-phase molecular biology reactions into the nanosensor and combining with nanoscale electrophoresis for read out and identification of the products. In addition, we will incorporate into the nanosensor in-plane pores for label-free detection of solid-phase products. Figure 7a shows preliminary results using dual in-plane nanopores for the detection and identification (TOF) of a ssDNA fragment. Finally, we will also replace the NIL process to fabricate the device with nano-injection moulding, which will allow for higher scale production at lower cost compared with NIL. A preliminary result of nano-injection moulding the nanosensor is shown in Figure 7b.

## Supplementary Material

Supplementary Material

## Impact Statement

We report a mixed-scale fluidic chip made from a plastic using a combination of photolithography (microstructures) and focused ion beam milling (nanostructures) to build a Si master from which could be generated a resin stamp via UV-nanoimprint lithography (NIL) and subsequent thermal NIL into a plastic to build the device. The chip consists of a series (8) of pillar arrays combined with nanochannels that are used to detect single-stranded DNAs (ssDNAs). Unique to this manuscript is the ability to use a combination of hydrodynamic and electrokinetic pumping to allow for passive valving. For hydrodynamic pumping, fluid is transported uniformly through the pillar arrays, but not through the nanochannels due to their high fluidic resistance. On the other hand, during electrokinetic pumping, ssDNAs are selectively introduced into the nanochannels due to the large electric field drop in these channels. The operation of the chip was verified using simulation and experimental results.
