# Supplementary material for: Fluidic operation of a polymer-based nanosensor chip for analysing single molecules
Source: Flow (Camb). Author manuscript; Available in PMC 2022 Aug 6. (PMC9356744; doi:10.1017/flo.2022.8)
Supplement: Supplementary Material [file NIHMS1821371-supplement-Supplementary_Material.zip › Revised ESI_Nanosensor_03272022.docx]

**Electronic Supplementary Information**

**Fluidic Operation of a Polymer-based Nanosensor Chip for Analyzing Single Molecules**

Swarnagowri Vaidyanathan,^1,4^ Sachindra Gamage,^2,4^ Kavya Dathathreya,^2,4^ Renee Kryk,^3,4^ Anishkumar Manoharan,^2,4^ Zheng Zhao,^1,4^ Lulu Zhang,^2,4^ Junseo Choi,^4,5^ Daniel Park,^4,5^ Sunggook Park,^4,5^ and Steven A. Soper^1,2,3,4,6*^


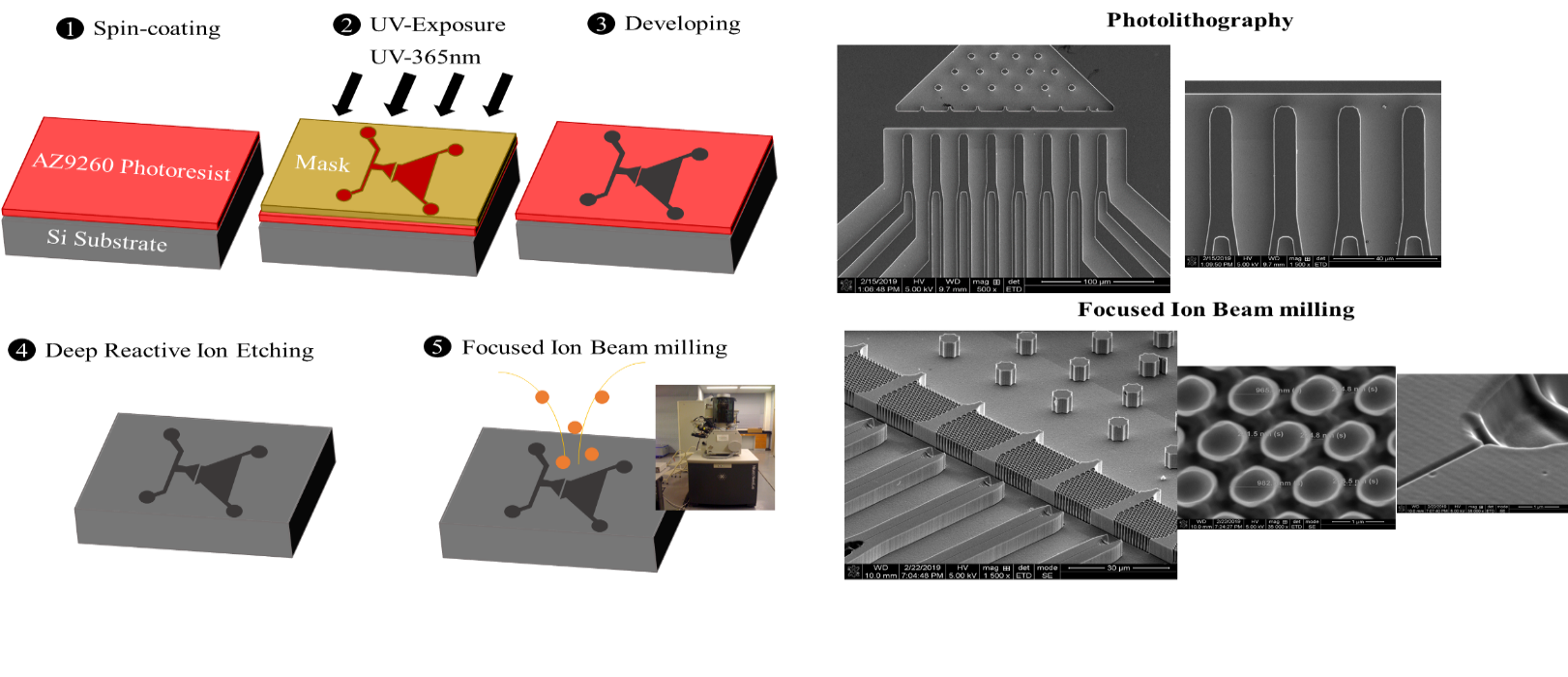


**A**

**B**

**C**

**i**

**ii**

**iii**

**i**

**ii**

**Figure S1**. (**A**) Schematic showing the process of fabrication of the Si master. Photolithography using AZ9260 resist was performed in 3 steps: (1) Spin coating of the AZ9260 resist onto the Si wafer; (2) exposure via a dark field mask to UV light (365 nm); and (3) resist development in MIF 300 developer. The photolithography was followed by DRIE (4), where the Si master was etched to a depth of 6-7 μm. Finally, FIB was used to generate the nanostructures onto the Si master to fabricate the pixel arrays and the flight tubes (5). (**B**) SEMs showing the Si master after photolithography and DRIE. Shown is an overview of the entire nanosensor (i) and placement holders for the flight tubes (ii). Not present are the pillar arrays and flight tubes, which were fabricated using FIB in a subsequent step. (**C**) SEMs of the Si master following FIB milling (i), where the desired nanostructures were produced, which included both the pixel arrays (ii) and the flight tubes (iii).


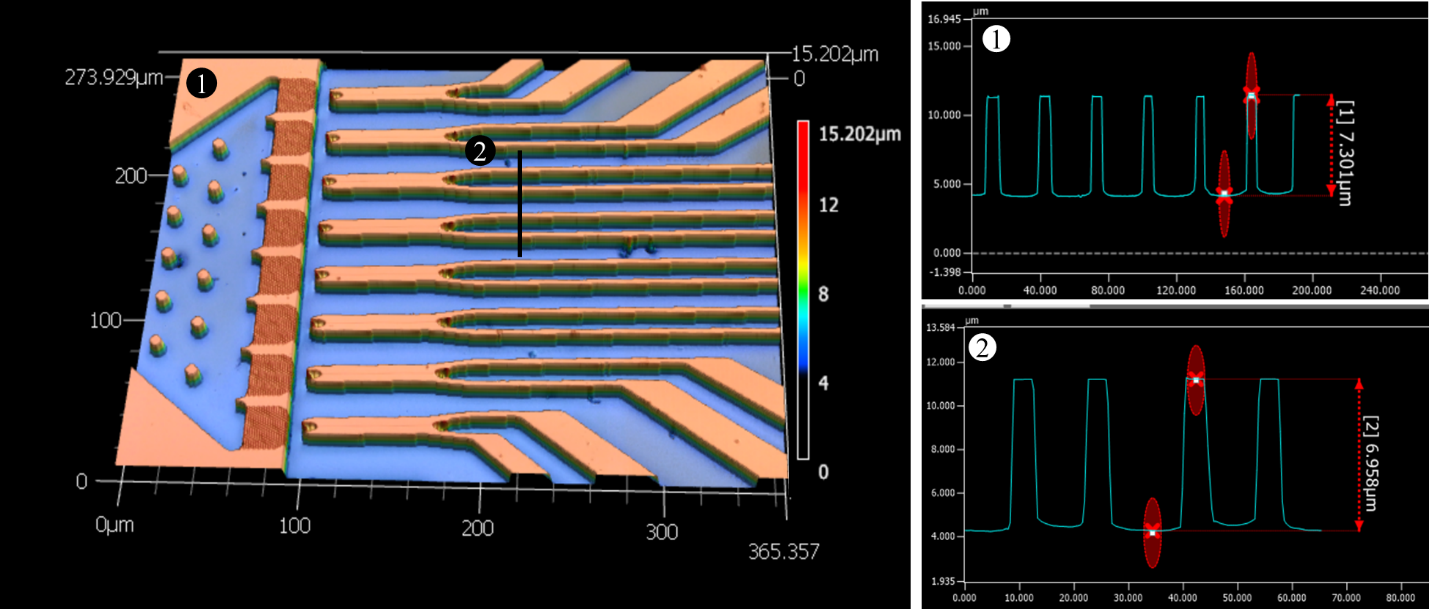


**A**

**B**

**Figure S2.** (**A**) Metrology of the nanosensor chip as determined by a rapid scanning confocal microscope (Keyence) showing the Si master following DRIE (to make the microstructures) and FIB milling (to make the nanostructures). (**B**) Line graph showing the depth across the baffle area, which was estimated to be ~7.3 μm (1). A line graph showing the depth across the microchannels that are placed after the flight tubes. The depth in this region was estimated to be ~6.9 μm (2).


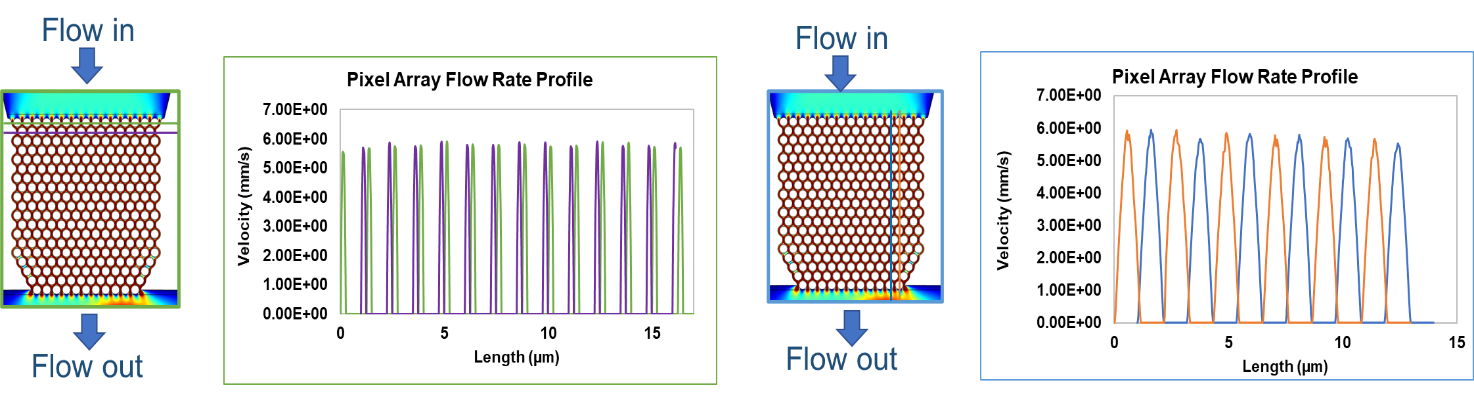


**Figure S3.** Schematic of the pixel arrays that were set up in COMSOL. Each array measured 20 µm x 20 µm with 268 pillars that were 1 µm in diameter with a 250 nm spacing. The nanosensor chip possessed 8 pixel arrays. (**A**) The linear velocity across two rows in one pixel array in the horizontal direction is represented, which were nearly similar (~5.7 mm/s) for all 8 pixel arrays (data not shown for other pixel arrays). (**B**) The pixel array flow rate profile across two rows of a single pixel array in the flow-in to flow-out direction, which showed uniform velocity (~5.7 mm/s) throughout the pixel array.

**B**

**A**


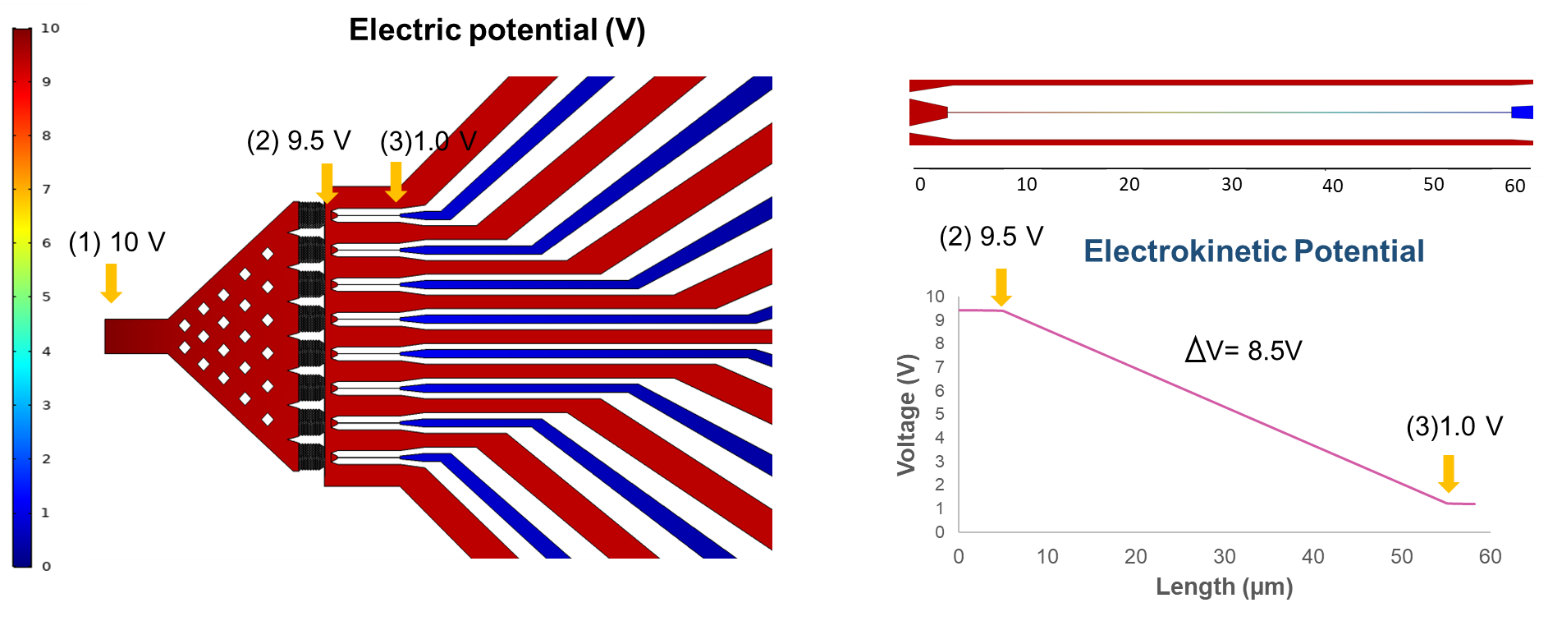


**Figure S4.** (**A**) COMSOL simulation of the electric potential throughout the nanosensor device. A potential of 10 V was applied across the device. The arrows depict the potential at both the entrance and exit of the nano-flight tube. (**B**) Heat map and line graph showing the potential drop across the nanometer flight tube. The line graph shows the drop in potential from the inlet of the nanosensor to the ends of the nanochannel, which is kept at earth ground. The 10 V was applied from the entrance point of the nanosensor, where it drops to 9.5 V after the pixel array. There was an 8.5 V drop across the nanometer flight tube, which measured 50 x 50 nm (width and depth) and was 50 µm in length.

**B**

**A**
