# Supplementary figures and images for: Fluidic operation of a polymer-based nanosensor chip for analysing single molecules
Source: Flow (Camb). Author manuscript; Available in PMC 2022 Aug 6. (PMC9356744; doi:10.1017/flo.2022.8)

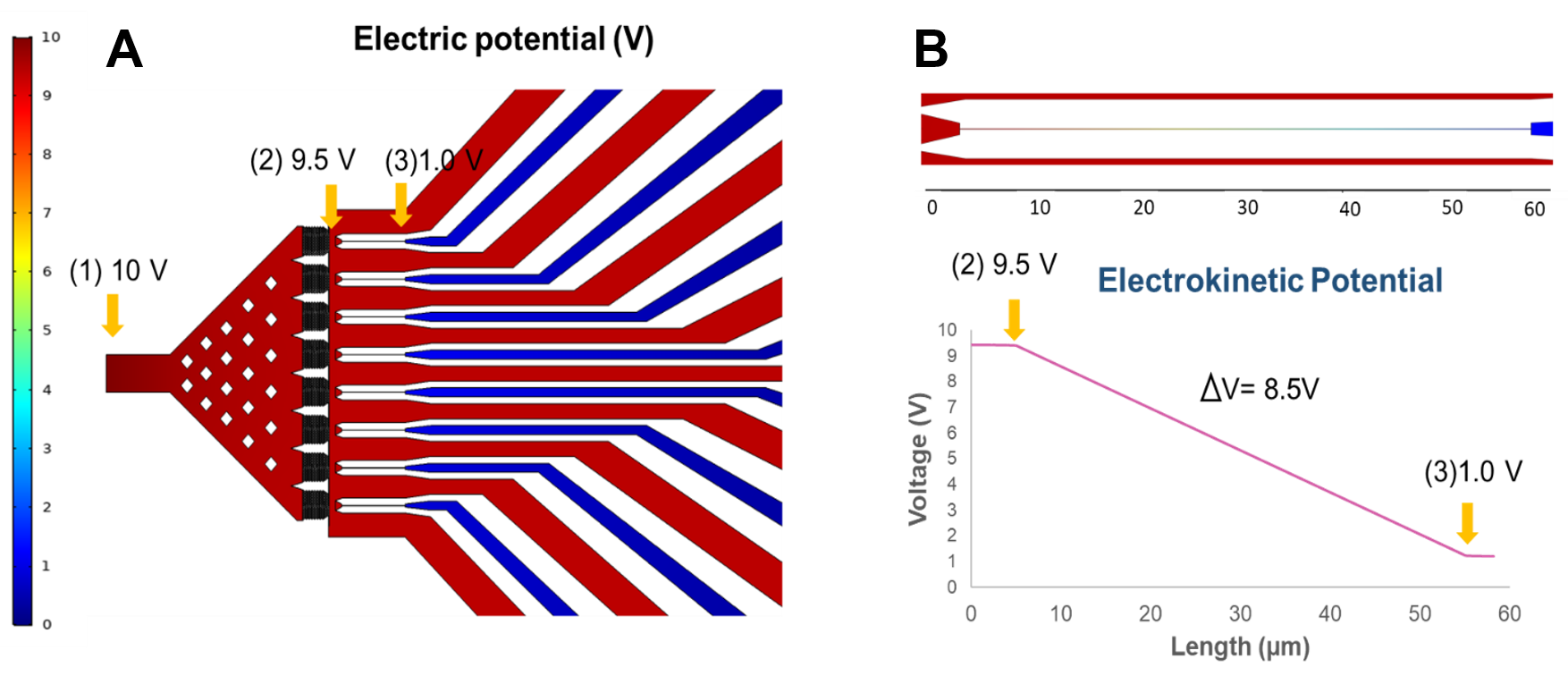

Supplement: Supplementary Material [file NIHMS1821371-supplement-Supplementary_Material.zip › Figure S4.tif]

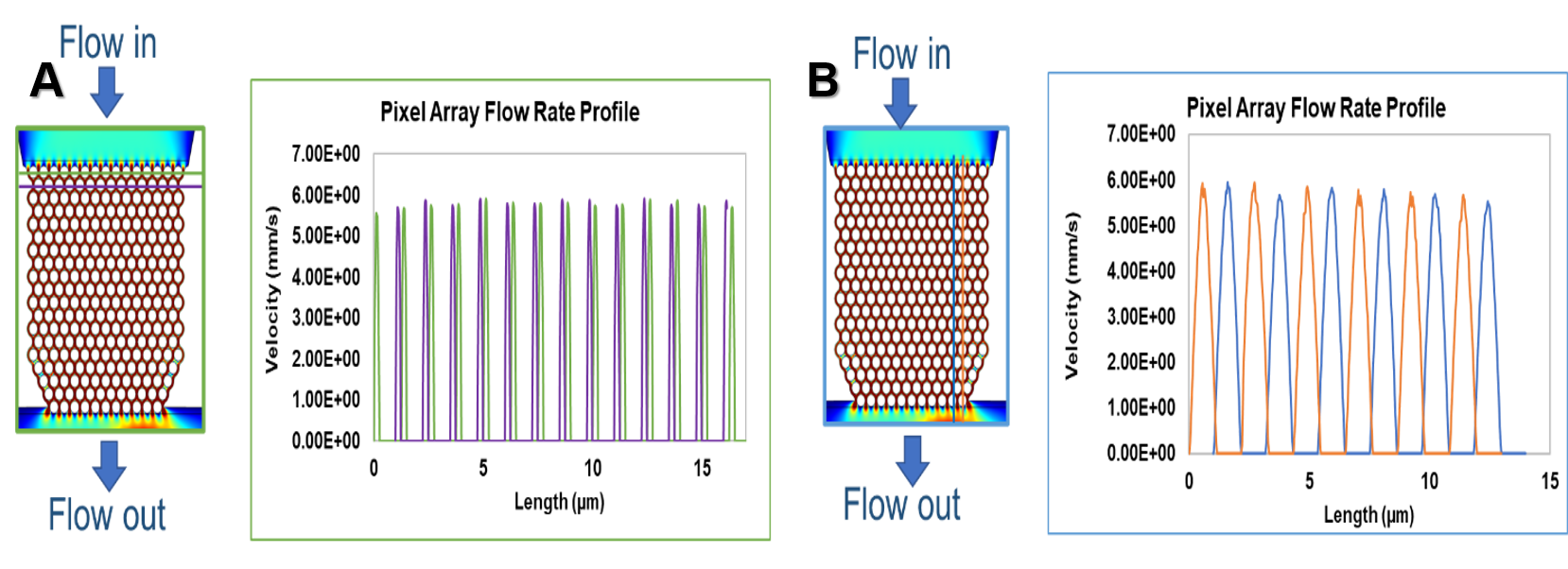

Supplement: Supplementary Material [file NIHMS1821371-supplement-Supplementary_Material.zip › Figure S3.tif]

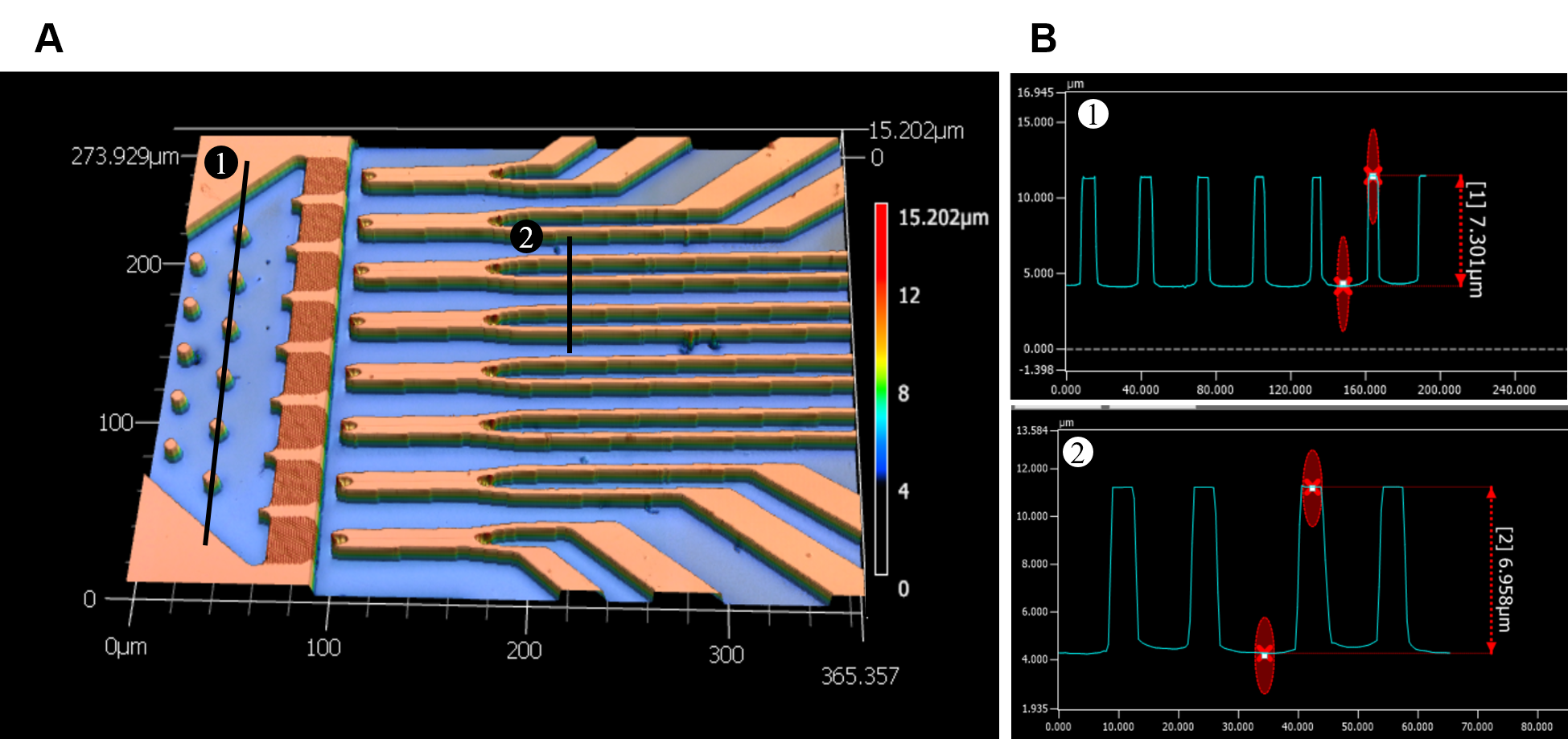

Supplement: Supplementary Material [file NIHMS1821371-supplement-Supplementary_Material.zip › Figure S2.tif]

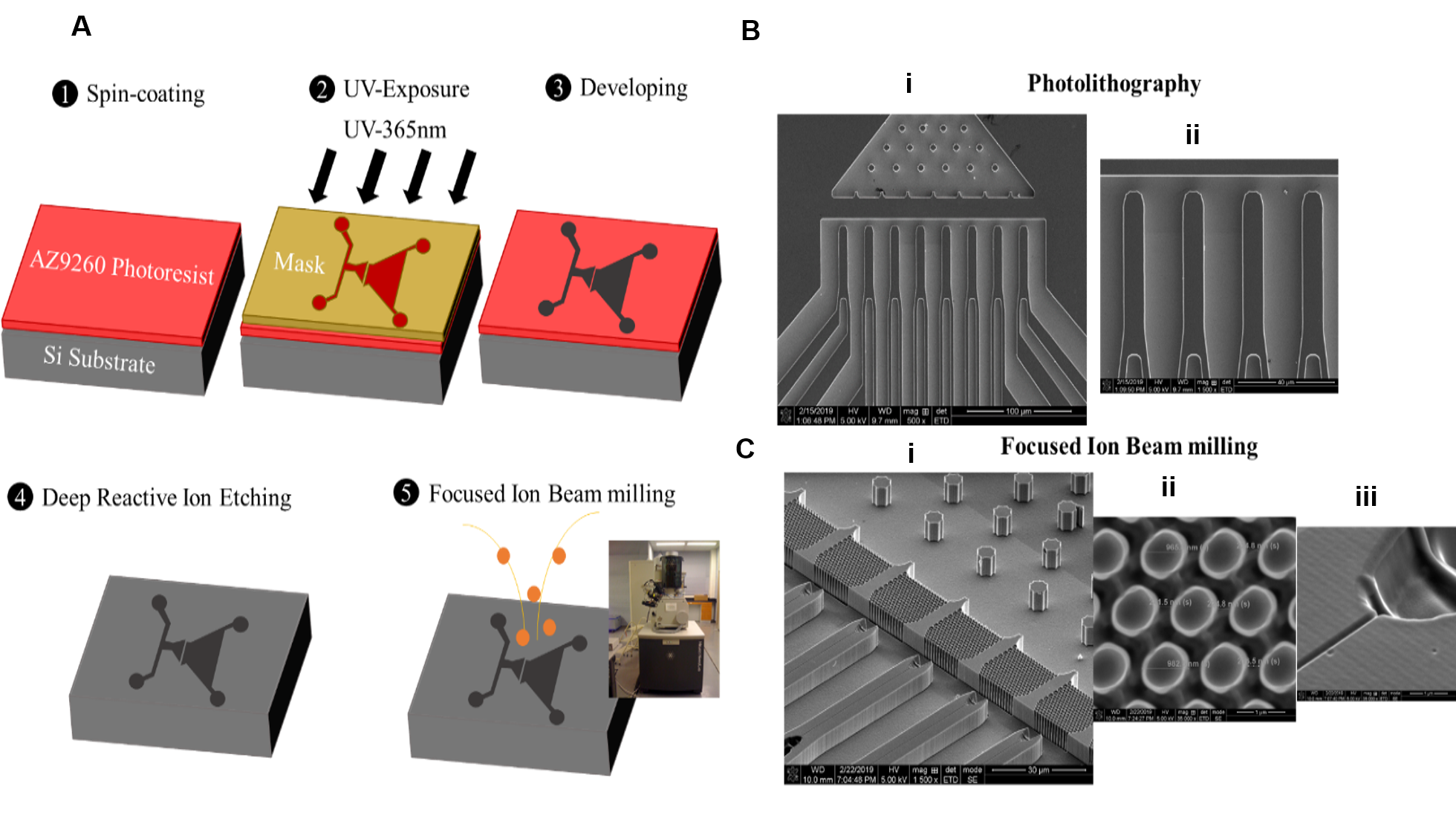

Supplement: Supplementary Material [file NIHMS1821371-supplement-Supplementary_Material.zip › Figure S1.tif]
